# The Manchester Triage System in optimizing triage in adult general medical emergency patients: the Triage Project

**DOI:** 10.1186/cc13257

**Published:** 2014-03-17

**Authors:** D Steiner, A Kutz, AC Rast, S Haubitz, L Faessler, M Batschwaroff, U Buergi, B Mueller, P Schuetz

**Affiliations:** 1 Kantonsspital Aarau, Switzerland

## Introduction

Some patients presenting to the emergency department (ED) currently face inacceptable delays in initial treatment due to suboptimal initial triage. Triage scores, such as the Manchester Triage

System (MTS), have not been well validated in unselected medical patients. Herein, we performed a prospective cohort study to assess the prognostic potential of the MTS and the prognostic biomarker pro- adrenomedullin (ProADM) to identify patients at high initial treatment priority, patients with admission to the ICU, and patients who die within a 30-day follow-up.

## Methods

This is a prospective, observational cohort study including all consecutive medical patients seeking ED care between June 2013 and October 2013, except nonadult and nonmedical patients. We collected detailed clinical information including the initial MTS and measured ProADM levels on admission in all patients. Initial treatment priority was adjudicated by two independent, blinded physicians based on all available results at the time of ED discharge to the medical ward. To assess outcomes, data from electronic medical records were used and all patients were contacted by telephone 30 days after hospital admission. The prognostic performance of MTS and ProADM was assessed in multivariate regression models with area under the receiver operating curve (AUC) as an overall measure of discrimination.

## Results

We included a total of 1,452 patients (58% males, mean age 66.6 years). A total of 20.1% (*n *= 292) were classified as high treatment priority, 5.4% (*n *= 79) were admitted to the ICU and 4.4% (*n *= 64) died within 30 days. The initial MTS showed a good prognostic accuracy to predict treatment priority (AUC 0.75) and ICU admission (AUC 0.76), but not for mortality prediction (AUC 0.58). Initial ProADM levels were independent predictors for all three outcomes and significantly improved the MTS score to AUCs of 0.78 for treatment priority, 0.80 for ICU admission and 0.84 for mortality.

## Conclusion

Within this large cohort of consecutive unselected medical patients seeking ED care, the MTS instrument in combination with a prognostic biomarker (ProADM) allowed accurate initial risk assessment in regard to treatment priority, ICU admission and mortality. A combined score has the potential to significantly improve initial risk assessment in patients, which may translate into faster and more targeted care and better clinical patient outcomes.

